# Protein function annotation based on heterogeneous biological networks

**DOI:** 10.1186/s12859-022-05057-3

**Published:** 2022-11-18

**Authors:** Sai Hu, Yingchun Luo, Zhihong Zhang, Huijun Xiong, Wei Yan, Meiping Jiang, Bihai Zhao

**Affiliations:** 1 School of Computer Science and Engineering, Changsha University, Changsha, 410022 Hunan China; 2 Department of Ultrasound, Hunan Provincial Maternal and Child Health Care Hospital, Changsha, 410008 Hunan China; 3 School of Mathematics, Changsha University, Changsha, 410022 Hunan China; 4 NHC Key Laboratory of Birth Defect for Research and Prevention, Hunan Provincial Maternal and Child Health Care Hospital, Changsha, 410100 Hunan China; 5 Hunan Provincial Key Laboratory of Industrial Internet Technology and Security, Changsha University, Changsha, 410022 Hunan China

**Keywords:** Protein function prediction, Heterogeneous biological network, Network propagation

## Abstract

**Background:**

Accurate annotation of protein function is the key to understanding life at the molecular level and has great implications for biomedicine and pharmaceuticals. The rapid developments of high-throughput technologies have generated huge amounts of protein–protein interaction (PPI) data, which prompts the emergence of computational methods to determine protein function. Plagued by errors and noises hidden in PPI data, these computational methods have undertaken to focus on the prediction of functions by integrating the topology of protein interaction networks and multi-source biological data. Despite effective improvement of these computational methods, it is still challenging to build a suitable network model for integrating multiplex biological data.

**Results:**

In this paper, we constructed a heterogeneous biological network by initially integrating original protein interaction networks, protein-domain association data and protein complexes. To prove the effectiveness of the heterogeneous biological network, we applied the propagation algorithm on this network, and proposed a novel iterative model, named Propagate on Heterogeneous Biological Networks (PHN) to score and rank functions in descending order from all functional partners, Finally, we picked out top *L* of these predicted functions as candidates to annotate the target protein. Our comprehensive experimental results demonstrated that PHN outperformed seven other competing approaches using cross-validation. Experimental results indicated that PHN performs significantly better than competing methods and improves the Area Under the Receiver-Operating Curve (AUROC) in Biological Process (BP), Molecular Function (MF) and Cellular Components (CC) by no less than 33%, 15% and 28%, respectively.

**Conclusions:**

We demonstrated that integrating multi-source data into a heterogeneous biological network can preserve the complex relationship among multiplex biological data and improve the prediction accuracy of protein function by getting rid of the constraints of errors in PPI networks effectively. PHN, our proposed method, is effective for protein function prediction.

## Background

Proteins are the basic organic matter that constitutes all cells and tissues of the living body. Accurately and automatically annotation of protein function is one of the fundamental tasks of bioinformatics, and it has become very hot in recent years. Methods for experimentally determining protein function such as gene expression inhibition [1], targeted mutation [2] and gene knockout [3] require considerable time and cost, and can only deal with one gene or protein at a time. With the increasing number of functional proteins to be labelled, such low-throughput experimental techniques cannot meet practical needs. Consequently, the computational method serves as a more suitable solution for determining protein function.

The rapid developments of high-throughput technologies have generated huge amounts of high-quality, large-scale protein interaction data, which provide fundamental and abundant data for network-based approaches to deduce protein functions. Schwikowski et al. [4] found that proteins interacting with each other generally share the same function, and proposed a method named NC for function prediction based on interacting neighbour voting. Chua et al. [5] proposed a functional similarity measurement method to recalculate the interaction strength of proteins by comprehensively utilizing the global structure of the protein interaction network characterized by direct and indirect neighbours, and improved the NC method on this basis. Since PPI networks can be represented by graph models, graph-theoretic algorithms were naturally applied to protein function prediction as well. Functions were deduced by the global connectivity pattern of the protein physical network, which was determined by minimizing the number of protein interactions between different functional categories [6]. The GLIDER [7] method predicted protein functions from a new graph-based similarity network instead of the PPI network. It can infer missing connections in PPI networks based on local and global graph properties.

Considering the incompleteness of the protein–protein interaction network, researchers combined multiple biological data with the protein interaction network to establish functional similarity networks for function annotation. Through statistical analysis, Liang et al. [8] found that two proteins are likely to perform the same or similar function if they have the same domain composition. Consequently, they constructed the Protein Overlap Network (PON) for protein function annotation. Peng et al. established the protein interaction network, domain co-occurrence network and functional interrelationship network and ran the random walk algorithm on these networks to deduce the function of proteins [9]. Sarker et al. reconstructed the protein interaction network using protein-domain association data and proposed the *GrAPFI* [10, 11] method to predict functions for the target protein by using the label propagation algorithm on this network. Generally speaking, if two genes or genes products have similarities in some context, we can conclude that they have the same or similar annotation terms. Song et al. determined the functions of the unknown protein by exploring its functional partner with the highest domain context similarity derived from their direct neighbours [12]. The DCS (Domain Combination Similarity) [13] calculated domain context similarity by adding domains of the protein itself and improved the performance of prediction of protein function. DeepGOPlus [14] deduced protein functions for the target protein based on the sequence similarity with known functions using deep learning techniques.

Despite effective improvement of these computational methods for function annotation, it is still a challenge to build an appropriate network model for the integration of multiplex biological data and PPI networks. The most prevalent way is to merge multiplex biological data into a single and unique network, in which the role of different types of data is reflected in the form of setting weights or parameters. Therefore, the parameter is an important factor that affects the performance of methods for function prediction, which generally depends on experience or the result of association analysis. Even if the parameter setting model is optimized, different species and even different data sets have different settings. So, how to set the parameter value will become one of the biggest barriers to the application of these prediction models. In addition, the construction of a single network ignores the differences among multiplex biological data and covers up the inherent attributes of different types of biological data. In this paper, we constructed a heterogeneous biological network with the integration of PPI networks and multiple biological data, including protein complexes and protein-domain association data. On this basis, we design a novel protein function prediction method named PHN (Propagate on Heterogeneous Networks) by applying the propagation algorithm [15] on the heterogeneous biological network. To evaluate the performance of PHN, we apply our method on the *Saccharomyces cerevisiae* PPI network. Experimental results show that the PHN method outperforms seven competing methods for prediction of protein function: NC [4], Song [12], DCS [13], DSCP [13], NPF [15], PON [8] and *GrAPFI* [10].

## Methods

**Fig. 1:**
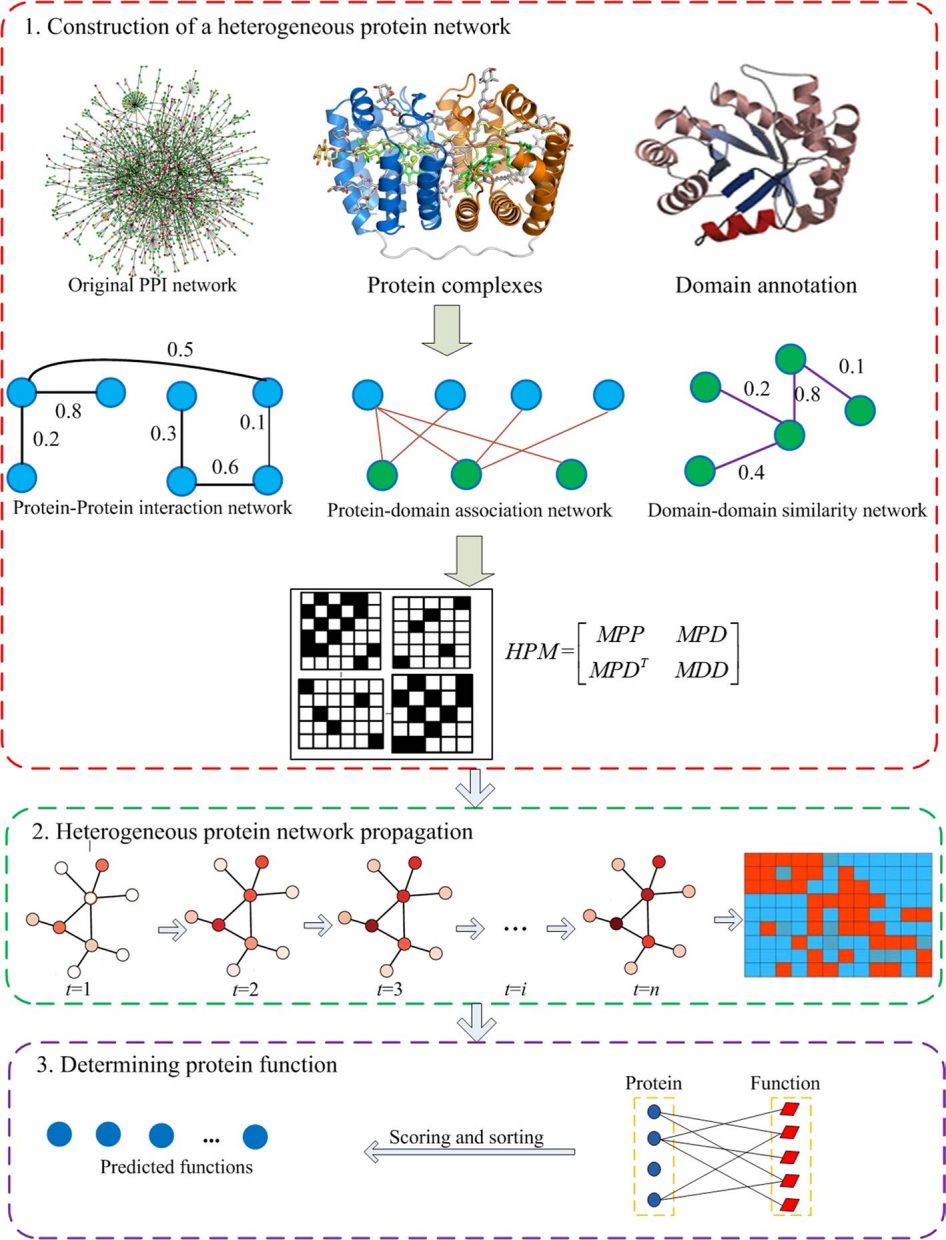
The flowchart of PHN method. **1** Constructing the heterogeneous biological network based on original PPI network, domain annotation and protein complex information. **2** Given a testing protein *u*, running the propagation algorithm on the heterogeneous biological network to obtain the functional similarity score vector *pr* between *u* and the other proteins. **3** Scoring and ranking functions in descending order coming from all functional partners and selecting the first *L* of them as candidates to annotate the target protein

The outline for the proposed PHN method includes (1) constructing a heterogeneous biological network by integrating the topology of PPI networks, protein-domain association data, and protein complex information, (2) running the propagation algorithm on the heterogeneous biological network to generate a functional similarity partners list for the given target protein, and (3) scoring and ranking functions from the partners list in descending, and picking out top *L* of them to annotate the unknown protein. The flowchart for the PHN method is provided in Fig. 1.

### Construction of a heterogeneous biological network

#### Protein–protein interaction network

It has been observed that more than 70% of proteins perform at least one function with their direct neighbours in networks [16]. We make a statistical analysis of yeast PPI data and observe that 21.3% of proteins share no functions with their direct neighbours, and they display enormous function similarity with some of their level-2 neighbours. In this paper, we evaluate functional similarity between a protein and its neighbours from the two different levels. Given a protein *u*, S_1_ and S_2_ denote the set of direct neighbours and level-2 neighbours of *u*, respectively. We classify all proteins except *u* into four categories: direct neighbours that are also level-2 neighbours (i.e. S_1_ ∩ S_2_), direct neighbours that are not level-2 neighbours (i.e. S_1_-S_2_), level-2 neighbours that are not direct neighbours (i.e. S_2_-S_1_) and protein that are not direct neighbours or level-2 neighbours (i.e.$\overline{{S_{1} \cup S_{2} }}$). In this work, the parameter α (0 < α < 1) is adopted to evaluate the importance of direct neighbours in functional analysis based on network topology. Accordingly, the functional similarity between nodes of the sets S_1_-S_2_ and *u* is defined asα. In particular, if a protein appears in both S_1_ and S_2_ (S_1_ ∩ S_2_), it is considered to have a necessary functional association with *u* and the functional similarity between them was set to 1. So, the functional similarity between a node in the sets S_2_-S_1_ and *u* is assigned 1 − α. Given a protein *v* in the network, the functional similarity between *u* and *v* based on the local topology of PPI networks can be defined as follow:

1 $$fs(u,v) = \left\{ {\begin{array}{*{20}l} {1,} \hfill & {\quad if\quad v \in S_{1} \cap S_{2} } \hfill \\ {\alpha ,} \hfill & {\quad if\quad v \in S_{1} - S_{2} \, } \hfill \\ {1 - \alpha ,} \hfill & {\quad if\quad v \in S_{2} - S_{1} } \hfill \\ {0,} \hfill & {\quad otherwise} \hfill \\ \end{array} } \right.$$

**Fig. 2:**
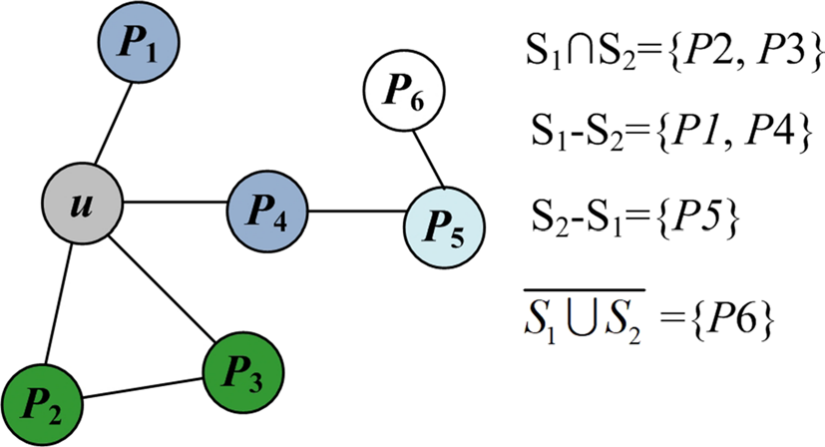
Example to illustrate four sets of protein pairs. Exhibition four sets of neighbour pairs of the target protein *u*, including S_1_ ∩ S_2_, S_1_–S_2_, S_2_–S_1_ and $\overline{{S_{1} \cup S_{2} }}$

Figure 2 illustrates these four sets of protein pairs. We are able to calculate the functional similarity between these neighbours and the target protein *u* according to Eq. 1: *fs*(*u*, *P*_1_) = α, *fs*(*u*, *P*_2_) = 1, *fs*(*u*, *P*_3_) = 1, *fs*(*u*, *P*_4_) = α, *fs*(*u*, *P*_5_) = 1 − α, *fs*(*u*, *P*_6_) = 0.

Protein complexes are functional units of macro-molecular organization consisting of interacting proteins that perform cellular biological functions [17]. Considering the fact that associated experimental techniques may cause a false positive result in protein–protein interaction assays, we apply protein complex data to evaluate the module similarity between proteins for the construction of a more reliable protein interaction network. Let *ms*(*u*, *v*) denote the module similarity of the interaction between *u* and *v*. Then, the module similarity of interaction (*u*, *v*) is calculated using the following equations:

2 $$ms(u,v) = \frac{{|C_{u} \cap C_{v} |^{2} }}{{|C_{u} | \times |C_{v} |}}$$

where *C*_*u*_ and *C*_*v*_ denote the set of protein complexes containing *u* and *v*, respectively. *C*_*u*_ ∩ *C*_*v*_ denotes the set of protein complexes containing both *u* and *v*. Finally, we construct a weighted protein–protein interaction network with high confidence by integrating the topology of PPI networks and protein complexes. The weight between protein *u* and *v* in the newly constructed protein–protein interaction network is the linear combination of their functional similarity and module similarity, and is defined as:

3 $$mpp(u,v) = \beta *fs(u,v) + (1 - \beta )*ms(u,v).$$

#### Protein-domain association network

Domain refers to the dense spherical region of protein subunit structure, which is composed of 100–200 amino acid residues, each with unique spatial conformation and different biological functions. If protein *u* contains domain *d*, *u* connects domain *dn* with an edge in the protein-domain association network and *mpd*(*u*, *dn*) = 1, otherwise, there is no edge between them and *mpd*(*u*, *dn*) = 0.

#### Domain-domain similarity network

In this work, we evaluate the similarity between domains through their associated protein interaction information. The domain-domain similarity network is constructed based on the above constructed weighted protein–protein interaction network and the protein-domain association network. Let *PL*(*dn*) represents the list of proteins that contain the domain *dn*. We quantitatively analyse the association between protein *u* and *PL*(*dn*) of the domain *dn* according to weighted protein interaction data, which is expressed as follows:

4 $$S\_PD(u,PL(dn)){ = }\mathop {\max }\limits_{{pn_{i} \in PL(dn)}} (mpp(u,pn_{i} ))$$

Then, for a given pair of domain *dn*_*i*_ and domain *dn*_*j*_, the similarity between them can be calculated as follows:

5 $$mdd(dn_{i} ,dn_{j} ) = \frac{{\sum {_{{pn_{x} \in PL(dn_{i} )}} S\_PD(pn_{x} ,PL(dn_{i} )) + \sum {_{{pn_{y} \in PL(dn_{j} )}} S\_PD(pn_{y} ,PL(dn_{j} ))} } }}{{|PL(dn_{i} )| + |PL(dn_{j} )|}}$$

where *PL*(*dn*_*i*_) and *PL*(*dn*_*j*_) are the protein set containing domain *dn*_*i*_ and domain *dn*_*j*_, respectively and $S\_PD(pn_{x} ,PL(dn_{i} ))$ denotes the association between protein *pn*_*x*_ and the set of protein $PL(dn_{i} )$.${|}PL(dn_{i} ){|}$ and ${|}PL(dn_{j} ){|}$ is the size of $PL(dn_{i} )$ and $PL(dn_{j} )$, respectively.

Based on the newly established protein–protein interaction network, protein-domain association network and domain-domain similarity network, a heterogeneous biological network is constructed and formally expressed by the adjacency matrix as follows:

6 $$HBM{ = }\left[ {\begin{array}{*{20}c} {MPP} & {MPD} \\ {MPD^{T} } & {MDD} \\ \end{array} } \right]$$

where *MPP*, *MPD* and *MDD* is the adjacency matrix corresponding to the protein–protein interaction network, protein-domain association network and domain-domain similarity network, respectively. *MPD*^*T*^ is a transport matrix of the matrix *MPD*.

### Heterogeneous biological network propagation

Restricted to the small-world and scale-free features of the protein–protein interaction network, existing distance measures such as shortest distance and Euclidean distance are not suitable for evaluating the functional similarity between proteins [18, 19]. In order to prioritize functional partners in the network for a target unknown protein, the propagation algorithm is run on the heterogeneous biological network. Firstly, we established a transition probability matrix *HBM*_*T* based on the matrix *MPD* by normalized operation, which is formalized as follows:

7 $$HBM\_T{ = }\left[ {\begin{array}{*{20}c} {MPP\_T} & {MPD\_T} \\ {MPD\_T^{T} } & {MDD\_T} \\ \end{array} } \right]$$

The transition probability from protein *pn*_*i*_ to protein *pn*_*j*_ is expressed as:

8 $$mpp\_t(i,j) = p(pn_{j} |pn_{i} ) = \left\{ {\begin{array}{*{20}l} {mpp(i,j)/\sum {_{j} mpp(i,j)} ,} \hfill & {if\quad \sum {_{j} mpd(i,j) = 0} } \hfill \\ {(1 - \lambda )mpp(i,j)/\sum {_{j} mpp(i,j)} ,} \hfill & {otherwise} \hfill \\ \end{array} } \right.$$

The transition probability from domain *dn*_*i*_ to domain *dn*_*j*_ is expressed as:

9 $$mdd\_t(i,j) = p(dn_{j} |dn_{i} ) = \left\{ {\begin{array}{*{20}l} {mdd(i,j)/\sum {_{j} mdd(i,j),} } \hfill & {if\quad \sum {_{j} mpd(j,i) = {0}} } \hfill \\ {(1 - \lambda )mdd(i,j)/\sum {_{j} mdd(i,j)} ,} \hfill & {otherwise} \hfill \\ \end{array} } \right.$$

The transition probability from protein *pn*_*i*_ to domain *dn*_*j*_ is expressed as:

10 $$mpd\_t(i,j) = p(dn_{j} |pn_{i} ) = \left\{ {\begin{array}{*{20}l} {\lambda mpd(i,j)/\sum {_{j} mpd(i,j)} ,} \hfill & {if\sum {_{j} mpd(i,j) \ne {0}} } \hfill \\ {0,} \hfill & {otherwise} \hfill \\ \end{array} } \right.$$

The transition probability from domain *dn*_*i*_ to protein *pn*_*j*_ is expressed as:

11 $$mpd\_t(j,i) = p(pn_{j} |dn_{i} ) = \left\{ {\begin{array}{*{20}l} {\lambda mpd(j,i)/\sum {_{j} mpd(j,i)} ,} \hfill & {if\quad \sum {_{j} mpd(j,i) \ne {0}} } \hfill \\ {0,} \hfill & {otherwise} \hfill \\ \end{array} } \right.$$

The parameter λ is the moving probability of the movement from the weighted protein–protein interaction network to the domain-domain similarity network and is assigned as 0.2 [20]. And then, we perform an iteration operation to calculate aggregated functional similarity scores between the given target protein *u* with other proteins by the following equation:

12 $$pr^{t + 1} = (1 - \gamma )*HBM\_T*pr^{t} + \gamma *pr^{0}$$

The parameter $\gamma \in [0,1][0,1]$ is balanced between the propagation information and initial scores, which is set to 0.5 [21, 22]. $pr^{0} { = }[h(P); \, h(D)]$ denotes the initial functional similarity score vector, which is derived from the protein–protein interaction network corresponding to the matrix *MPP* and protein-domain association network. For a given protein *p*_*i*_, its initial functional similarity score between the target protein *u* is expressed by the weight of interaction between *p*_*i*_ and *u* in the protein–protein interaction network, that is:

13 $$h(p_{i} ) = mpp(u,p_{i} )$$

As for domains, their initial functional similar scores are derived from scores of their relevant proteins. Given a domain *d*_*j*_, its initial score is computed by the following formula:

14 $$h(d_{j} ) = \mathop {\max }\limits_{{p_{x} \in PL(d_{j} )}} (h(p_{x} ))$$

where *PL*(*d*_*i*_) is the protein set of domain *d*_*i*_. In Eq. (12), if $\left\| {pr^{t + 1} - pr^{t} } \right\|_{{1}} \ge \varepsilon$, then *t* = *t* + 1 and return to the previous step to continue the iteration, otherwise, the iteration end. When the propagation converges, we can obtain an aggregated scores vector *pr*, in which proteins are arranged in descending according to their functional similarity to the target protein *u*.

### Determining protein function

MethodS typified by Song et al. [11] assigned all functions of the protein with the highest similarity value to the target protein with unknown functions. However, our statistical results on recent PPI data indicate that the function overlaps of more than half of protein pairs fell into [0.4, 0.6] and that of only 11.99% of protein pairs is above 0.6. So, functions are scored and ranked in descending order coming from all functional partners and the top *L* of them are picked out as candidates to annotate the target protein in this work. Let *FN* = {*fn*_1_, *fn*_2_,…, *fn*_*m*_} be a list of distinct functions of proteins in *pr* that have a functional similarity score greater than 0 to the target protein *u*. For a given function *fn*_*i*_ in *FN*, its ranking score is obtained using the following formula:

15 $$S(fn_{i} ) = \sum\limits_{j = 1}^{n} {pr(j)} *t_{ij}$$

In Eq. (15), if *pn*_*i*_ contains function *fn*_*i*_, then *t*_*ij*_ = 1, otherwise *t*_*ij*_ = 0. The parameter *L* is assigned the number of functions of the protein within *pr*, which has the highest functional similarity score to the target protein *u*. Algorithm 1 gives the overall framework of the proposed PHN method.
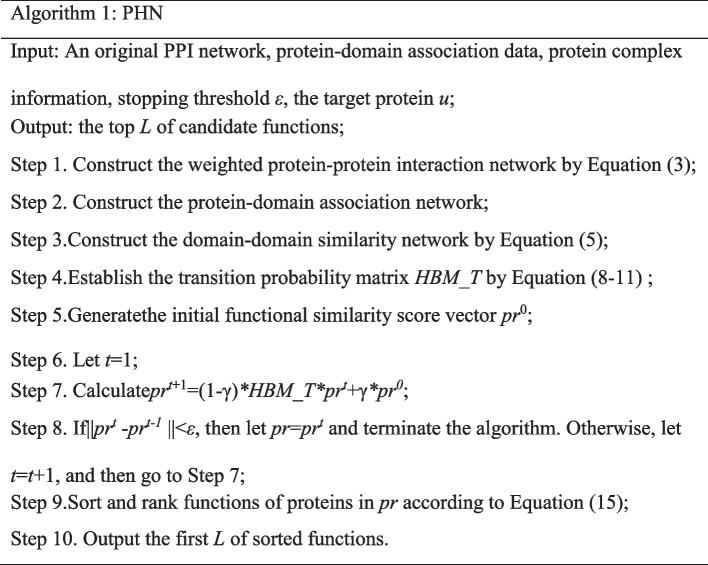


## Results and discussion

### Experimental data

**Fig. 3:**
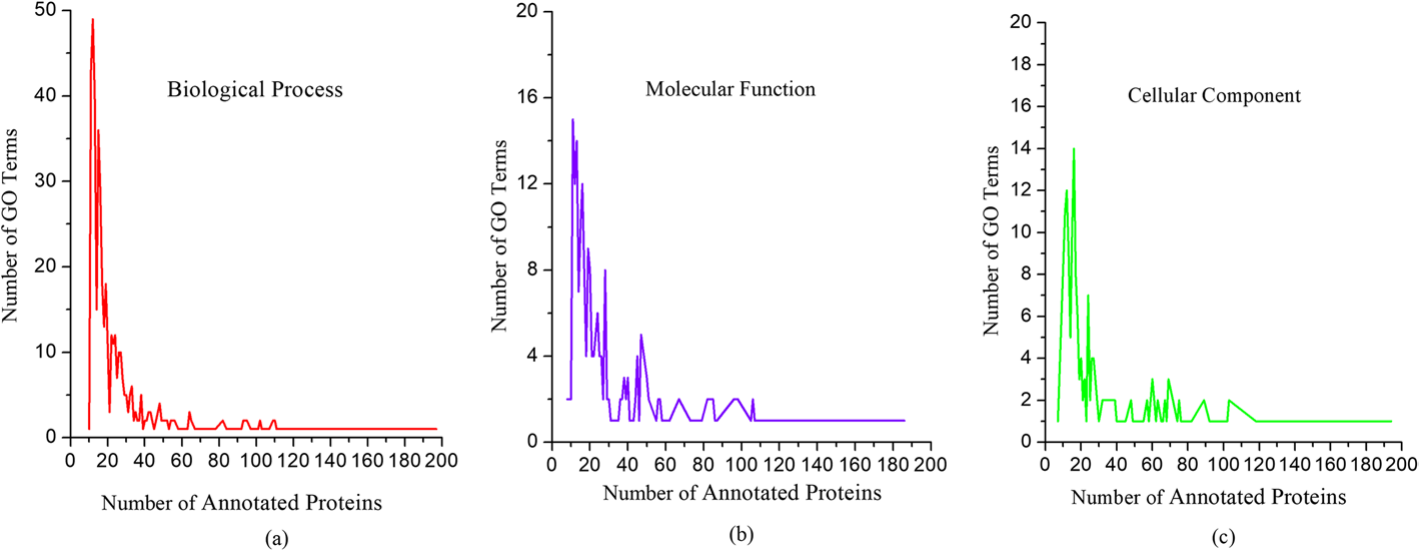
Distribution of GO term in the BioGRID dataset. This Figure shows the distribution of GO term in the BioGRID dataset. X-axis represents the number of annotated protein. Y-axis represents the number of GO terms. **a** Biological process, **b** molecular function, **c** cellular component

**Fig. 4:**
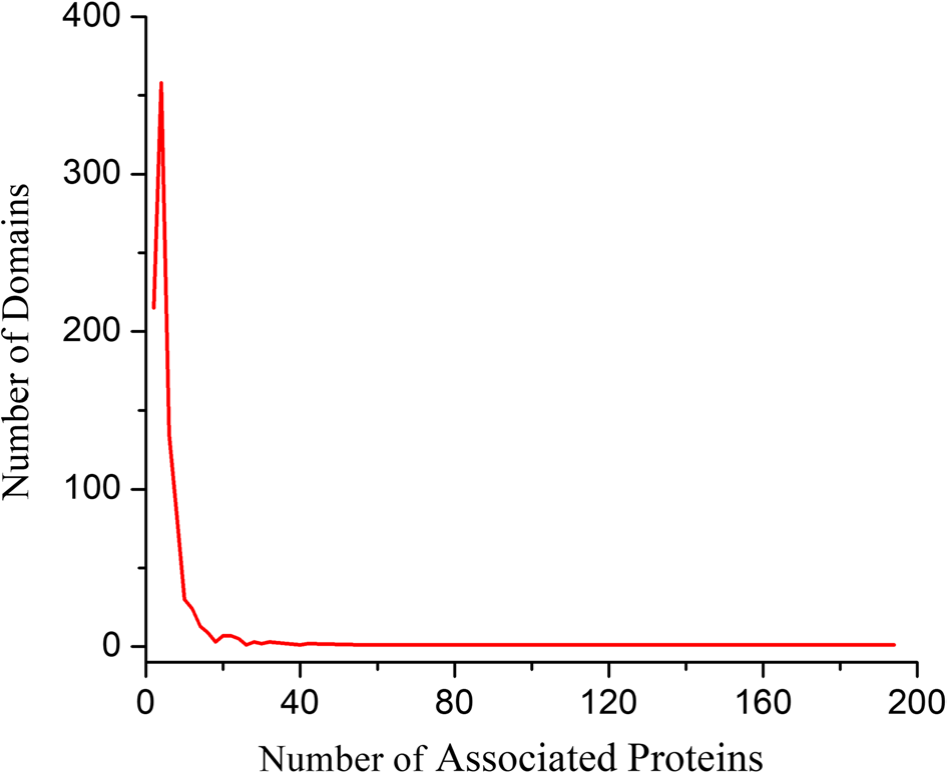
Distribution of domain in the BioGRID dataset. This Figure shows the distribution of domain in the BioGRID dataset. X-axis represents the number of associated protein. Y-axis represents the number of domains

The *Saccharomyces cerevisiae* (yeast) protein interaction networks are widely used in the research of network-based function prediction algorithms as a gold standard data because of their completeness and convincement. Here, we apply PHN and seven competing methods on yeast interaction networks to test the performance of our new method. The original protein interaction data used in this work is downloaded from the BioGRID (Biological General Repository for Interaction Datasets) database [23], compiled on Aug. 25, 2022. The BioGRID PPI network contains 3145 proteins and 15,070 experimentally detected interactions with self-interactions and repeated interactions removed. The experimentally detected protein complex set for construction of the weighted PPI network is obtained from the CYC2008 database [24], which consists of 408 complexes involving 1408 proteins in the BioGRID database. The function annotation of proteins used for validation is downloaded from the Gene Ontology Consortium (GOC) [25]. The GO terms maintain three structured controlled vocabularies, which describe gene products in terms of their associated biological processes (BP), cellular components (CC) and molecular functions (MF). In the BioGRID network, 2957, 2250 and 2130 out of 3145 proteins are annotated by BP, MF and CC, respectively. The gold standard GOC consists of 518, 219 and 174 GO terms for BP, MF and CC respectively. Figure 3 depicts the distribution of GO terms in BP, MF and CC, respectively. We obtain 4936 protein-domain association data with invalid and duplicate relationships removed from the PFAM [26] database. It involves 906 distinct domain types related to 2044 proteins of the PPI networks. Figure 4 shows the distribution of Domain types in the BioGRID network. Figure 4 reveals that more than 63% of the domain types are associated with less than 5 proteins.

### Evaluation criteria

In this work, the performance of PHN is evaluated by two cross validations, including leave-one-out cross validation and ten-fold cross validation. To measure the quality of predicted functions by our method, we adopt the ROC (Receiver-Operating Curve) [27, 28] as an assessment. The ROC curve is plotted with FPR (False Positive Rates) and TPR (True Positive Rates) [29], which is widely used in performance evaluation for protein function prediction. In addition, we also calculate the Precision, Recall and F-measure of the proposed PHN method. Precision is the fraction of predicted functions that are matched with benchmark functions while Recall is the fraction of benchmark functions that are matched with predicted functions. As the harmonic mean of precision and recall, F-measure is another assessment to evaluate the performance of the protein function prediction method synthetically.

### Effect of parameter β

On the construction of the weighted protein–protein interaction network, we employ a user-defined parameter β to balance functional similarity and module similarity in Eq. 3. With different values of parameter β, the performances of prediction might differ greatly. As a result, we investigate the effect of parameter β on the PHN method by running 11 times with equal intervals of β from 0 to 1. The corresponding values of Recall, Precision and F-measure at different values of β are calculated. Table 1 shows how these performance evaluation criteria of our method fluctuate under various values of β based on GO terms in BP, MF and CC. From Table 1, we can easily see that the comprehensive evaluation criteria F-measure of PHN in BP reached the maximum value when β is assigned to 0.8. Similarly, the PHN method obtains optimal performance in MF and CC when β is set to 0.7 and 0.9, respectively. Therefore, we set the default value of parameter β in BP, MF and CC to 0.8, 0.7 and 0.9 respectively in all the following experiments.

**Table 1:** Effect of parameter β on the performance of HPN

	0	0.1	0.2	0.3	0.4	0.5	0.6	0.7	0.8	0.9	1
BP											
Recall	0.301	0.511	0.514	0.518	0.521	0.525	0.529	0.530	**0.536**	0.537	0.135
Precision	0.309	0.492	0.494	0.497	0.501	0.508	0.510	0.512	**0.516**	0.513	0.104
F-measure	0.305	0.501	0.504	0.507	0.511	0.516	0.519	0.521	**0.526**	0.525	0.118
MF											
Recall	0.441	0.547	0.549	0.550	0.554	0.552	0.555	**0.556**	0.549	0.545	0.238
Precision	0.446	0.551	0.553	0.554	0.556	0.557	0.558	**0.561**	0.561	0.561	0.239
F-measure	0.443	0.549	0.551	0.552	0.555	0.554	0.557	**0.559**	0.555	0.553	0.238
CC											
Recall	0.556	0.588	0.589	0.592	0.599	0.604	0.606	0.607	0.607	**0.612**	0.175
Precision	0.551	0.576	0.579	0.584	0.590	0.593	0.592	0.595	0.596	**0.598**	0.181
F-measure	0.554	0.582	0.584	0.588	0.594	0.598	0.599	0.601	0.602	**0.605**	0.178

Bold values represent the optimal value of parameter β set in BP, MF and CC

### Leave-one-out cross-validation

**Fig. 5:**
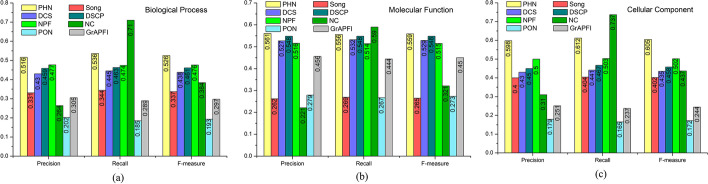
The predicted results of various methods using leave-one-out cross validation. Numbers of each bar are the values for each score, including average Precision, Recall and F-measure. **a** Biological process, **b** molecular function, **c** cellular component

In this part, we use leave-one-out cross validation to evaluate the quality of the functions predicted by PHN and seven other competing methods. In each round, there is only one protein in the testing set and the rest in the training set. Firstly, we evaluate the comprehensive performance of PHN and seven other competing algorithms, such as NC, Song, DCS, DSCP, NPF, PON and *GrAPFI* by the average Precision, Recall and F-measure. Figure 5 shows the overall performance of the above eight methods in the matter of Precision, Recall and F-measure. PHN is the only method with F-measure above 50% in BP, MF and CC. Compared with NC, a classic network-based function prediction method, the F-measure of PHN for MF, CC and BP category is improved by 36.98%, 74.14% and 38.44% respectively. While compared with NPF which is the latest proposed function prediction algorithm with protein domain and complex information integrated, PHN also shows remarkable performance. Particularly, for GO terms in CC, the F-measure of PHN is 20% higher than that of NPF. Figure 5 indicates that PHN obtains the highest prediction precision of all the methods and the second-highest recall after NC. The recall of PHN is inferior to that of NC, due to the functions annotation strategy that PHN only selects the top part of the predicted functions to annotate the unknown protein, while the NC method assigns all the functions of neighbours to the target protein. This treatment of the NC method causes a lot of noise to emerge in its predicted functions, resulting in a sharp drop in precision. In this experiment, the recall of NC for the BP, MF and CC category is 32.31%, 6.11% and 20.38% higher than that of PHN, respectively. While its precision in BP, MF and CC is 95.83%, 154.1% and 92.49% lower than that of PHN.

**Fig. 6:**
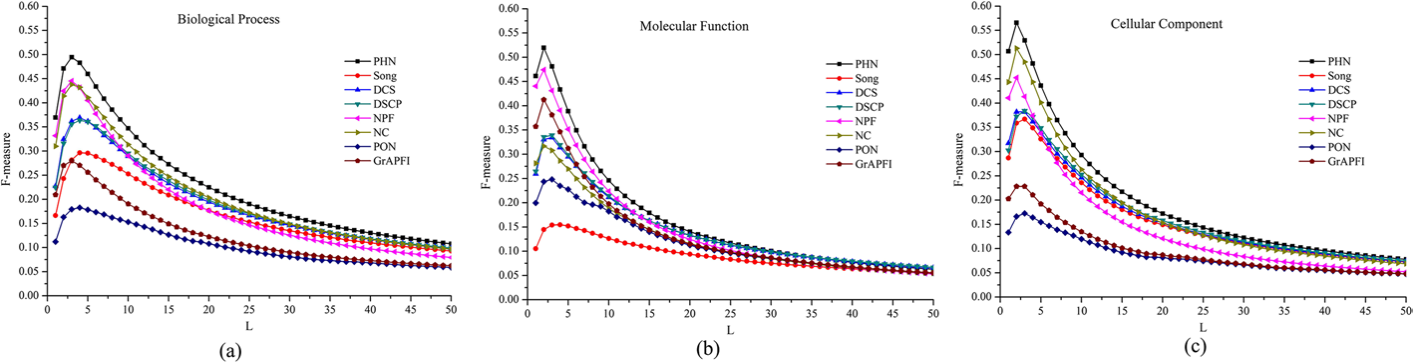
F-measure curves as the number of selected functions *L* varies. This Figure depicts the F-measure of our method and other competing methods fluctuate under various value of the parameter *L*. The X-axis and Y-axis represents of the curve are the values of the parameter *L* and average F-measure, respectively. **a** Biological process, **b** molecular function, **c** cellular component

The choice of the number of functions predicted by various methods is an important factor affecting their performance. We try our best to select a unified candidate functions selection strategy for each method to comprehensively and objectively compare and analyse the performance of different methods. The predicted functions are arranged in descending order based on the functional similarity score values derived by PHN, NPF, NC, PON and *GrAPFI*, respectively. And then top *L* of candidate functions are picked out to annotate the target protein. For the three methods of Song, DCS and DSCP, the top *M* (*M* ≤ *L*) of proteins with the highest function similarity to the target protein are selected, and the top *L* of functions from these *M* proteins are selected as predicted functions. A more valuable comparison between algorithms is presented by plotting F-measure curves as the value of *L* (*L* ≤ 50) varies. Figure 6 shows the F-measure of our method and other competing methods fluctuates under various values of *L* in BP, MF and CC. From Fig. 6, we can see that the setting of *L* in the interval [2, 5] is the optimal solution for all methods. The experimental results also show that PHN achieves the best performance of all methods, regardless of the value of *L*.

**Fig. 7:**
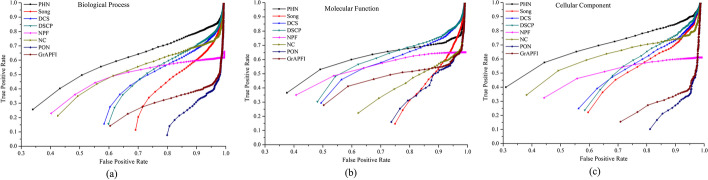
ROC curves of eight methods using leave-one-out cross validation. The figure denotes the ROC (receiver-operating) curves of PHN and other seven competing methods (NC, Song, DCS, DSCP, NPF, PON and *GrAPFI*) based on the average prediction performance over all testing proteins. The X-axis and Y-axis of the ROC curves are the values of false positive rate and true positive rate, respectively. **a** Biological process, **b** molecular function, **c** cellular component

Moreover, we also employ Receiver-Operating Curve (ROC) curves and the corresponding areas under the ROC curve (AUROC) values to evaluate the overall performance of each method. Firstly, functions are ranked in descending order according to the functional similarity scores to the target proteins computed by each method. After that, the top *K* functions are picked out and put into positive data set as candidate functions, and then the remaining functions are stored in negative data set. The upper limit values of *K* in BP, MF and CC are 518, 219, and 174, respectively. With different values of *K* selected, the values of TPR (False Positive Rates) and FPR (True Positive Rates) are computed for each method, respectively. Then, the values of TPR and FPR are plotted in ROC curves with different cut-off values. The experimental results are illustrated in Fig. 7. From Fig. 7, we can see intuitively that the ROC of PHN in BP, MF and CC is clearly above those of all other methods.

For further comparison, we calculate the AUROC of all these methods. For the BP category, the AUROC of PHN is 154.03%, 75.33%, 75.91%, 40.29%, 28.06%, 548.92% and 209.91% larger than that of Song, DCS, DSCP, NPF, NC, PON and *GrAPFI*, respectively. Compared with NC which has the best performance in ROC curves among seven competing methods, the AUROC of PHN for the MF and CC category is improved by 123.4% and 23.56%, respectively.

### Ten-fold cross validation

**Fig. 8:**
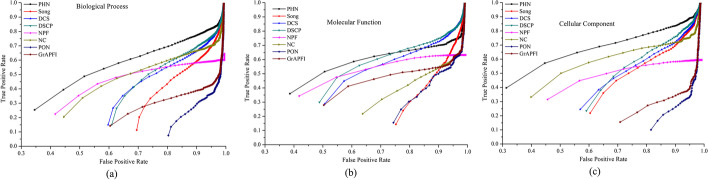
ROC curves of eight methods using ten-fold cross validation. This Figure shows the ROC curves of PHN and other seven methods using ten-fold validation. Proteins are randomly divided into ten equal sets, one set for testing, and the rest for training. And then, the cross validation is repeated process for ten rounds, using various folds as the training and validation data in each round. The results of ten folds are averaged to generate the final performance. **a** Biological process, **b** molecular function, **c** cellular component

TO avoid the possible deviation caused by leave-one-out cross validation, we further evaluate the prediction performance of the PHN method using the ten-fold cross validation. The entire set of proteins is divided into ten equal sets randomly, nine of which are used for training and the remaining part is used for testing. The process is repeated 1000 times, each time using another testing set. The results of ten folds are averaged to generate the final performance. Table 2 lists the prediction results of eight methods, including the average Precision, Recall and F-measure. Table 2 shows that PHN still performs the best, in terms of precision and F-measure. Taking the BP category as an example, the F-measure of PHN is 55.93%, 21.28%, 14.51%, 8.69%, 37.17%, 180.33% and 72.73% higher than that of Song, DCS, DSCP, NPF, NC, PON and *GrAPFI*, respectively. In addition, we plot ROC curves of all methods for the MF, CC and BP category as shown in Fig. 8. The AUROC of PHN in BP is 152.21%, 77.71%, 76.27%, 35.47%, 33.10%, 570.28% and 199.27% larger than that of Song, DCS, DSCP, NPF, NC, PON and *GrAPFI*, respectively. As for the MF and CC category, PHN increases the AUROC by no less than 15% and 28%, respectively, compared with other competitive comparison methods.

**Table 2:** The results of PHN and seven competing methods using ten-fold cross validation

Categories	Methods	Recall	Precision	F-measure
BP	PHN	0.523	0.504	0.513
	Song	0.335	0.323	0.329
	DCS	0.431	0.415	0.423
	DSCP	0.450	0.446	0.448
	NPF	0.470	0.475	0.472
	NC	0.678	0.259	0.374
	PON	0.175	0.192	0.183
	*GrAPFI*	0.289	0.306	0.297
MF	PHN	0.547	0.553	0.549
	Song	0.266	0.259	0.263
	DCS	0.522	0.519	0.520
	DSCP	0.536	0.536	0.536
	NPF	0.507	0.509	0.508
	NC	0.563	0.217	0.313
	PON	0.261	0.273	0.267
	*GrAPFI*	0.444	0.455	0.449
CC	PHN	0.604	0.584	0.594
	Song	0.398	0.394	0.396
	DCS	0.428	0.418	0.423
	DSCP	0.458	0.442	0.449
	NPF	0.503	0.555	0.520
	NC	0.706	0.304	0.425
	PON	0.160	0.175	0.167
	*GrAPFI*	0.237	0.251	0.244

## Conclusions

ProteinS are one of the most important and diverse macromolecules in cell life activities. Precise labelling of protein function information is important for promoting the research and development of protein mechanism analysis, disease mechanism analysis and control, new drug research and development, crop production promotion, bio-energy development and so on. The rapid developments of high-throughput technologies have generated large quantities of protein–protein interaction (PPI) data, which prompts the emergence of computational methods to determine protein function. Despite the effective improvement of these computational methods, building a suitable network model to integrate multiplex biological data remains a challenge due to the incomplete and error-prone raw PPI data. How to construct an effective network model that integrates multiplex biological data and network topology remains a challenge. Current methods aggregated multiple biological data into a single network, in which the role of different types of data is reflected in the form of setting weights or parameters. The choice of weighting parameters and the inherent properties of different biological data restrict the further development of these methods. In this work, we construct a heterogeneous biological network with two categories of nodes: protein and domain. To shake off the bound of the small-world and scale-free of PPI networks, innovatively, we use the propagation algorithm on the heterogeneous biological network and obtain a functional partners list with aggregated similarity to the target protein. Finally, we score and rank functions from the partners list in descending order. The number of candidate functions we selected is equal to the number of functions of the neighbour most similar to the target protein. To assess the overall performance of PHN, we use the leave-one-out cross validation and ten-fold cross validation. The F-measure and AUROC of our method improved by more than 17% and 15%, respectively, compared with other approaches. The experimental results also indicate that PHN is a specific and effective method that can predict protein function.

## Data Availability

Publicly available datasets are analysed in this study. This data and the PHN program can be found here: https://github.com/husaiccsu/PHN.
